# Regional assessment of carotid artery pulse wave velocity using compressed sensing accelerated high temporal resolution 2D CINE phase contrast cardiovascular magnetic resonance

**DOI:** 10.1186/s12968-018-0499-y

**Published:** 2018-12-20

**Authors:** Eva S. Peper, Gustav J. Strijkers, Katja Gazzola, Wouter V. Potters, Abdallah G. Motaal, Ilse K. Luirink, Barbara A. Hutten, Albert Wiegman, Pim van Ooij, Bert-Jan H. van den Born, Aart J. Nederveen, Bram F. Coolen

**Affiliations:** 1 Department of Radiology and Nuclear Medicine, Amsterdam UMC, University of Amsterdam, Amsterdam, Netherlands; 2 Department of Biomedical Engineering and Physics, Amsterdam UMC, University of Amsterdam, Amsterdam, Netherlands; 3 Department of Vascular Medicine, Amsterdam UMC, University of Amsterdam, Amsterdam, Netherlands; 4 Department of Neurology, Amsterdam UMC, University of Amsterdam, Amsterdam, Netherlands; 5 Image Guided Therapy, Philips Healthcare, Singapore, Singapore; 6 Department of Clinical Epidemiology, Biostatistics and Bioinformatics, Amsterdam UMC, University of Amsterdam, Amsterdam, Netherlands; 7 Department of Pediatrics Emma Children’s Hospital, Amsterdam UMC, University of Amsterdam, Amsterdam, Netherlands

**Keywords:** Arterial stiffness, Pulse wave velocity, Compressed sensing, Phase contrast MRI, Carotid atherosclerosis

## Abstract

**Background:**

Cardiovascular magnetic resonance (CMR) allows for non-invasive assessment of arterial stiffness by means of measuring pulse wave velocity (PWV). PWV can be calculated from the time shift between two time-resolved flow curves acquired at two locations within an arterial segment. These flow curves can be derived from two-dimensional CINE phase contrast CMR (2D CINE PC CMR). While CMR-derived PWV measurements have proven to be accurate for the aorta, this is more challenging for smaller arteries such as the carotids due to the need for both high spatial and temporal resolution. In this work, we present a novel method that combines retrospectively gated 2D CINE PC CMR, high temporal binning of data and compressed sensing (CS) reconstruction to accomplish a temporal resolution of 4 ms. This enables accurate flow measurements and assessment of PWV in regional carotid artery segments.

**Methods:**

Retrospectively gated 2D CINE PC CMR data acquired in the carotid artery was binned into cardiac frames of 4 ms length, resulting in an incoherently undersampled k_y_-t-space with a mean undersampling factor of 5. The images were reconstructed by a non-linear CS reconstruction using total variation over time as a sparsifying transform. PWV values were calculated from flow curves by using foot-to-foot and cross-correlation methods. Our method was validated against ultrasound measurements in a flow phantom setup representing the carotid artery. Additionally, PWV values of two groups of 23 young (30 ± 3 years, 12 [52%] women) and 10 elderly (62 ± 10 years, 5 [50%] women) healthy subjects were compared using the Wilcoxon rank-sum test.

**Results:**

Our proposed method produced very similar flow curves as those measured using ultrasound at 1 ms temporal resolution. Reliable PWV estimation proved possible for transit times down to 7.5 ms. Furthermore, significant differences in PWV values between healthy young and elderly subjects were found (4.7 ± 1.0 m/s and 7.9 ± 2.4 m/s, respectively; *p* < 0.001) in accordance with literature.

**Conclusions:**

Retrospectively gated 2D CINE PC CMR with CS allows for high spatiotemporal resolution flow measurements and accurate regional carotid artery PWV calculations. We foresee this technique will be valuable in protocols investigating early development of carotid atherosclerosis.

**Electronic supplementary material:**

The online version of this article (10.1186/s12968-018-0499-y) contains supplementary material, which is available to authorized users.

## Background

Cardiovascular magnetic resonance (CMR) has emerged as a powerful tool for non-invasive assessment of biomarkers indicating regional vessel wall diseases [1]. An important and early marker of vessel wall disease is arterial stiffness, which can be characterized by pulse wave velocity (PWV) [2]. PWV is the velocity at which the arterial pressure wave, created by cardiac contraction, travels through the arteries. PWV has been proven to be an independent predictor of cardiovascular events and atherosclerosis [3, 4]. Arterial stiffness increases with age, which has been demonstrated by an age-related increase in global PWV and regional aortic PWV [5, 6].

Severe atherosclerotic plaques are frequently observed at specific locations [7]. PWV measurements in the carotid arteries are of particular interest, since they are a major atherosclerosis-prone site associated with stroke [7–9]. Increased regional PWV was shown to precede atherosclerotic lesions even before intima-media thickening, another early marker for atherosclerosis [7]. Experimental results also demonstrate that vascular stiffening caused by early atherosclerosis is unequally distributed over the length of large vessels [10]. This implies that assessing heterogeneity of arterial stiffness by multiple local measurements of PWV might be more sensitive than global PWV to identify early atherosclerotic lesions. Therefore, to better understand the processes of regional plaque development and to improve early detection of local changes in arterial stiffness, measurements of regional PWV might prove essential.

In addition, arterial stiffness is not uniform in all vessels and PWV increases distal to the heart and further down the vascular tree. This emphasizes the relevance of regional PWV assessment. For instance, a decreasing aortic to carotid PWV ratio has been implicated in cognitive damage, since it allows the transmission of high blood pressure to the microvasculature of the brain [6, 11].

PWV values can be derived using CMR by acquiring time-resolved flow curves from two-dimensional CINE phase contrast CMR (2D CINE PC CMR) measurements at two different sites within the artery of interest. The time shift between the flow curves is the transit time of the arterial pulse wave. PWV is then defined as the distance between the sites divided by the transit time and is related to arterial stiffness through the Moens-Korteweg equation [2]. Compared to PWV measurement in the aorta [12], carotid PWV measurements are more challenging since the smaller lumen diameter (aorta 31–34 mm [12], carotid 4–6 mm [13]) and higher PWV [6] require a significant increase in spatial and temporal resolution. Nevertheless, carotid PWV measurements using cardiac triggered 2D PC CMR have successfully been performed by Kröner et al. [6] in arterial segments with a length of 15–20 cm. However, to measure PWV at even shorter distance, a further increase in temporal resolution is required because transit times decrease with the length of the arterial segment. Unfortunately, the spatiotemporal resolution in cardiac triggered PC CMR is limited by available scan time and lowest achievable repetition time (TR).

In previous studies, it was shown that an increased temporal resolution of cardiac gated sequences can be obtained by using retrospective gating and undersampling in combination with a compressed sensing (CS) reconstruction [13]. In short, retrospective gating involves continuous acquisition of data asynchronous to the electrocardiogram (ECG) signal. Due to small variations in the heart rate, phase encoding lines are therefore acquired at incoherent positions throughout the cardiac cycle [13]. This allows binning of the data into a high number of cardiac frames, although this leads to an undersampled k-space for each cardiac frame. Fortunately, CS reconstruction is highly efficient in exploiting temporal relations between frames and recovering information of undersampled data without significantly sacrificing image quality or the underlying temporal behavior [13, 14]. To our knowledge, this approach has never been applied in combination with 2D CINE PC CMR derived PWV measurements.

In this study, we developed a new clinical 3 T CMR protocol for high spatiotemporal resolution carotid artery 2D CINE PC CMR. The technique was validated using ultrasound and CMR phantom experiments. The ability of the technique to detect relevant differences in regional PWV was demonstrated in two groups of healthy young and elderly subjects.

## Methods

### Sequence design and retrospective undersampling

**Fig. 1:**
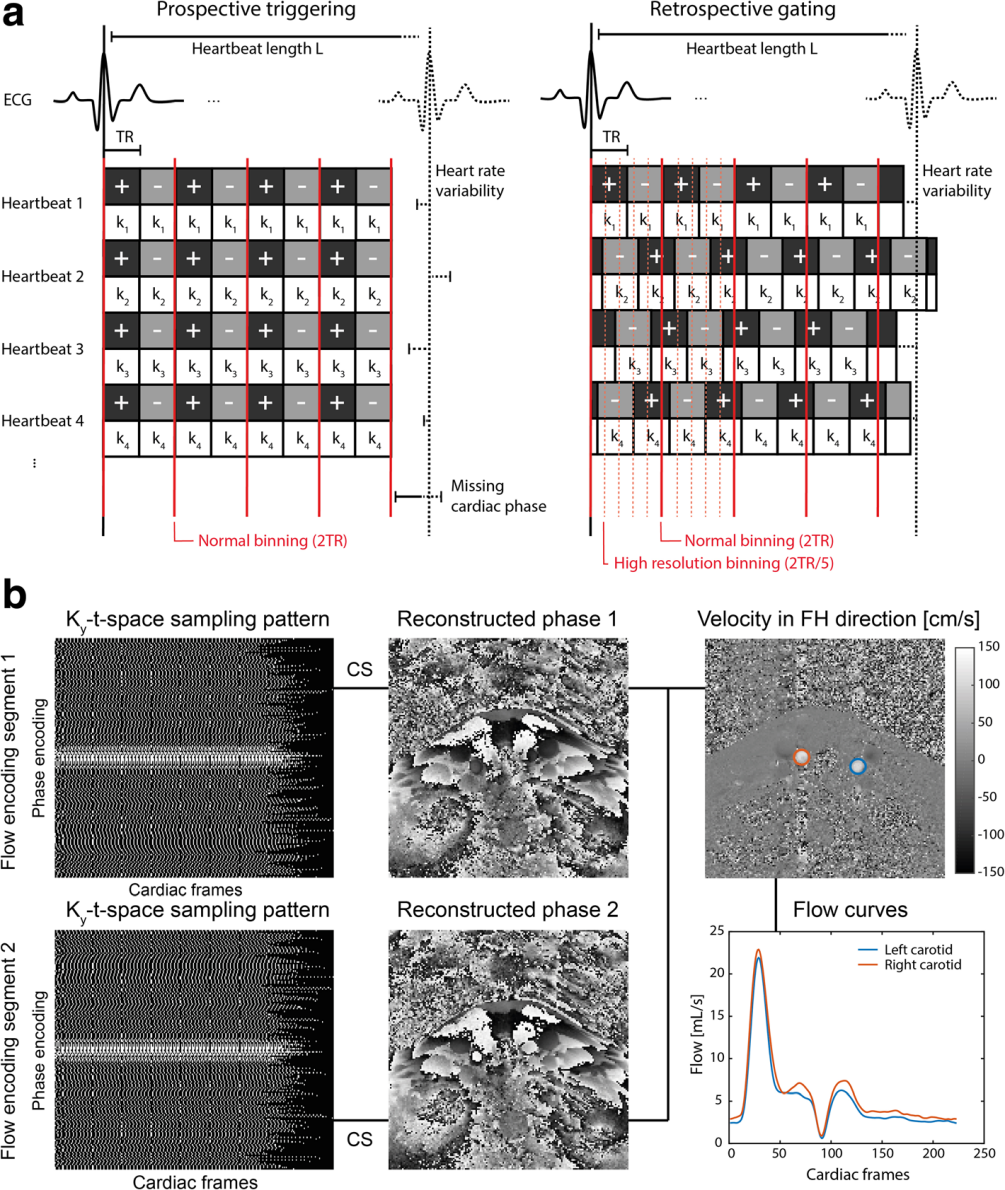
Principle of retrospectively-gated 2D CINE PC CMR. **a** Electrocardiogram (ECG) triggering for a 2D CINE phase contrast (PC) cardiovascular magnetic resonance (CMR) scan using prospective triggering and retrospective gating. In prospective triggering (left) the acquisition is started by the detection of the R-wave peak. This leads to the same time point of acquisition (the trigger time) for each phase encoding step (k_1_ to k_4_) and a uniform binning afterwards. The + and - symbols indicate the 2 encoding segments needed for flow quantification in the foot-head (FH) direction. In retrospective gating (right) every phase encoding step is repetitively sampled during the course of the heart beat asynchronously with the R-peak detection. Due to slight variations in the length of individual heartbeats, the trigger time varies. The data is binned into cardiac frames with a duration shorter than 2xTR, which creates a pseudo-random undersampling pattern. **b** Reconstruction pipeline. After binning the data in 4 ms cardiac frames, each flow encoding segment with a different undersampling pattern is reconstructed separately, after which the two phase images are combined into a velocity-encoded image. Region-of-interests (ROIs) are drawn around the left and right carotid arteries to quantify the flow

Figure 1 illustrates the principle behind the proposed method. For conventional prospective ECG triggered CINE scans, k-line acquisitions are performed at fixed intervals with respect to the R-peaks, which leads to identical timing of all cardiac frames within each measured cardiac cycle (Fig. 1a, left). Considering two flow encoding directions, the temporal resolution of each cardiac frame is then limited by two times the repetition time (TR).

For retrospectively gated CINE scans, acquisitions are performed asynchronously with the ECG. Consequently, due to natural heart rate variations, each acquisition takes place at a random time point within the cardiac cycle. The number of cardiac frames can be chosen retrospectively and, more importantly, can be higher than the number of TRs that fit within one cardiac cycle (Fig. 1a, right). While this results in a significant increase in temporal resolution of the resulting PC data, it also results in an undersampled k_y_-t-space. To overcome this, reconstruction of the incoherently undersampled data was performed using an iterative CS reconstruction algorithm as shown later. To further facilitate CS reconstruction, k_y_-lines were sampled more densely towards the k-space center ensuring sufficient filling of the center for all cardiac frames (Fig. 1b, left). For specifically enabling 2D CINE PC CMR scans, each k_y_-line was repeated using both positive and negative flow encoding gradients.

The proposed method was implemented on a whole-body 3 T CMR scanner (Ingenia, Philips Healthcare, Best, The Netherlands). For PWV measurements, two slices were acquired with a fast field echo (FFE) 2D PC CMR sequence, with unidirectional velocity encoding in the foot-head (FH) direction and the following parameters: VENC = 150 cm/s, TR = 8.0 ms, TE = 3.9 ms, FA = 8°, slice thickness = 3 mm, spatial resolution = 0.8 × 0.8 × 3 mm^3^, FOV = 130 × 130 mm^2^, acquisition matrix = 160 × 160 and acquisition time (2 slices) = 5 min. Scanning software was adapted in order to acquire custom defined variable density k-space trajectories ensuring that each cardiac frame had sufficient filling of the k-space center.

### Retrospective binning and compressed sensing reconstruction

Raw data was processed using MATLAB (MathWorks, Natick, Massachusetts, USA) and MRecon (Gyrotools LLC, Zurich, Switzerland). Retrospective binning of measured k_y_-lines into multiple cardiac frames was based on the R-top delays available from the raw data, which indicated the exact acquisition time point with respect to the detected R-peak. Importantly, we chose an ‘absolute’ binning strategy with a fixed cardiac frame length of 4 ms instead of a ‘relative’ binning strategy with a fixed number of cardiac frames (e.g. 250). This is to ensure that systolic frames of the cardiac cycle overlap between different cardiac cycles, despite possible changes in the R-R interval, which are mostly caused by shortening or lengthening of the diastolic phase [15].

After binning of the data, CS reconstruction of both magnitude and phase images was performed using the BART toolbox [16] (Fig. 1b). This was done independently for each of the two flow encoding segments. Specifically, a general CS reconstruction algorithm was used, described by the minimization function:

1 $$ \arg\ \min\ \left\{{\left\Vert {\mathrm{F}}_{\mathrm{U}}\mathrm{m}-\mathrm{y}\right\Vert}_2+\upalpha\ {\left\Vert \mathrm{Tm}\right\Vert}_1\right\} $$

with m and y being the reconstructed and measured data, respectively and F_U_ the undersampled Fourier operator. The l_2_ norm enforces data consistency between y and m. Although the data is defined in x,y-space and time, the sparsifying total variation (TV) operator T is only performed along the time dimension. Furthermore, α acts as a regularization parameter and was chosen to be 0.001. A non-linear conjugate gradient descending algorithm of 20 iterations was used to solve the minimization problem for all reconstructions. Reconstruction parameters (α and the number of iterations) were heuristically defined by minimizing the difference between flow curves of the CS reconstruction and flow curves of the fully sampled dataset of 20 ms temporal resolution. For this comparison the CS reconstructions (with a temporal resolution of 4 ms) were down-sampled to 20 ms.

### PWV calculation

PWV is calculated by dividing the distance between the two measured slices (Δx) by the transit time (Δt) between the corresponding flow curves (PWV = Δx/Δt). For the in vivo case, Δx was determined from coronal 3D black-blood images (see in vivo experiments*)*. The actual path length between the 2D CINE PC slices was determined by tracking the center of the vessel with OsiriX [OsiriX Foundation, Geneva, Switzerland] [17]. To generate the flow curves for each slice, firstly, phase difference images (=velocity images) were calculated from the CS reconstructed phase images from each flow segment. Subsequently, flow per cardiac frame was calculated by averaging all velocity values within a region of interest (ROI) and by multiplying that value with the area of this ROI (Fig. 1b, right).

**Fig. 2:**
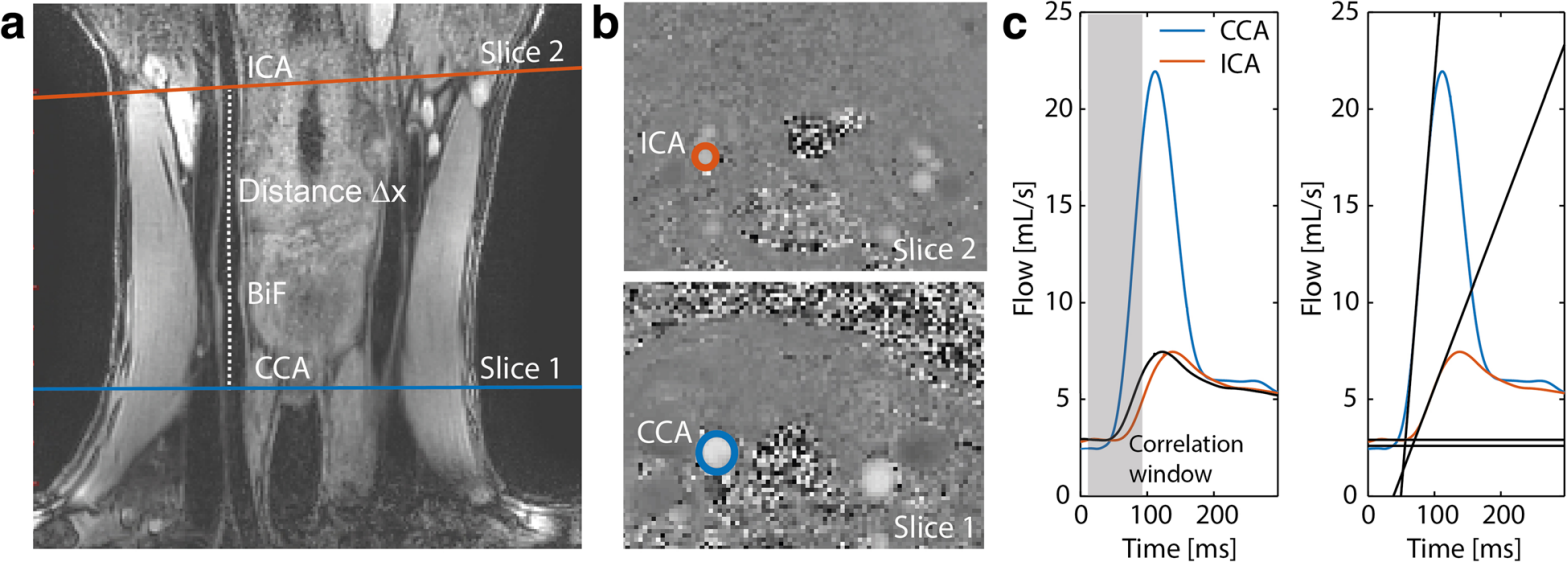
PWV calculation using 2D CINE PC CMR and black-blood CMR. **a** Black-blood image and curved path ∆x tracked between the two slices. **b** Velocity images acquired perpendicular to the common carotid artery (slice 1) and the internal carotid artery (slice 2). **c** The transit time was calculated from the two flow curves using the CC (left) and FF (right) method. Details on the CC and FF methods are shown in Additional file 1: Figure S1. CCA=common carotid artery; ICA=internal carotid artery

To determine the transit time between two flow curves, two distinct methods were used: 1) foot-to-foot (FF) method [18], which calculates the shift between the foots of the systolic flow peaks and 2) cross-correlation (CC) method [19], which finds the time shift that results in the best correlation between the flow curves (in the region of the systolic flow peak or the correlation window). For both methods, additionally a normalization of the flow curves and a sigmoidal fit through the systolic flow peak was applied. Figure 2 shows an overview of the PWV methods. A detailed description of the methods (including normalization and sigmoidal fitting) can be found in the Additional file 1: Figure S1. Because no consensus in literature exists on which method performs best, the averaged result of all analyses was used in order to provide a robust performance of PWV calculation [2].

### Flow phantom experiments

**Fig. 3:**
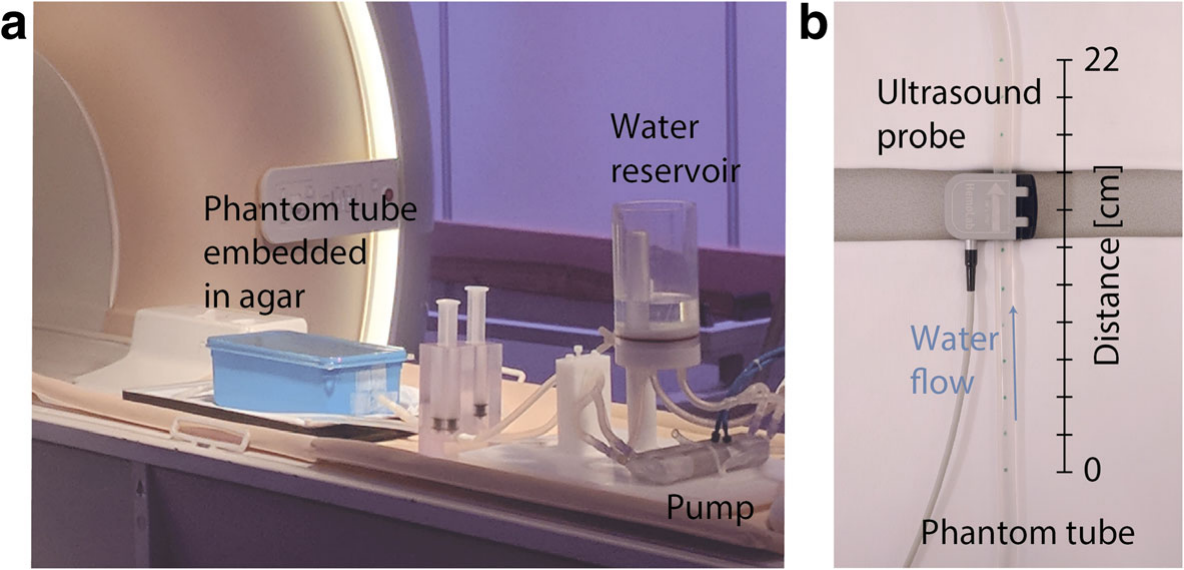
Phantom setup. **a** Flow phantom setup in the CMR scanner and (**b**) during the ultrasound measurement

A customized in vitro flow phantom (Fig. 3) was used to validate if our technique provides accurate flow curves at high temporal resolution, as well as accurate PWV values derived from the measured flow data. Results were compared to flow curves obtained from ultrasound probe measurements. The flow phantom (LifeTec Group BV, Eindhoven, The Netherlands), consisted of a silicon tube of 30 cm length, with an inner diameter h = 5 mm and a wall thickness = 2 mm similar to a human common carotid artery. A pulsatile water flow was created at a heart rate of 60 bpm with a simulated heart rate variability of 10% to facilitate incoherent sampling of k_y_-t-space. A temporal mean flow of 5 mL/s was established with a peak systolic flow rate of 24 mL/s. Flow was measured with an ultrasound probe (temporal resolution 1 ms) of 2 cm width that was clamped on the tube at 4 cm distance to the connectors and with an inter-slice distance of 2 cm at 12 positions along the tube. Ultrasound data was acquired for at least 30 heart beats per position and retrospectively binned (absolute binning) and averaged according to the signal of the pulse generator of the pump. In this way, ultrasound data consisted of an ensemble of heart beats, similar to CMR data. For the CMR experiment, the phantom tube was placed in an agar bath to ensure a stable phase offset correction afterwards. The agar bath was prepared with a relatively high amount of water to ensure sufficient flexibility of the phantom. 2D CINE PC slices were placed at equal positions as compared to those from the ultrasound acquisitions and were acquired perpendicular to the tube. The ECG signal was provided by the pulse generator of the pump. The data was retrospectively binned (absolute binning) in cardiac frames of 4 ms length.

### In vivo experiments

In vivo carotid artery PWV scans were performed in the right carotid artery of 23 young (30 ± 3 years, 12 [52%] women) and 10 elderly (62 ± 10 years, 5 [50%] women) healthy subjects using a dedicated 8-channel carotid coil (Shanghai Chenguang Medical Technologies, Shanghai, China). All procedures were approved by the local institutional review board (METC) of the Academic Medical Center in Amsterdam and were carried out according to the declaration of Helsinki. All participant gave written informed consent.

After scout scans, a coronal 3D black-blood iMSDE gradient echo sequence [20] was performed to visualize the carotid arteries (Fig. 2a). Then, retrospectively gated 2D CINE PC acquisitions were performed in two slices (distance ∆x = 7.8 ± 1.4 cm) perpendicular to the common and internal carotid artery. After acquisition, datasets from both slices were binned in cardiac frames of 4 ms length. PWV values were calculated as described above.

### Statistical analysis

Differences in PWV values between the two age groups were tested with a two-sided Wilcoxon rank-sum test. The statistical level of significance was set to 0.05. Statistical analysis was performed in MATLAB (Mathworks, Inc., Natick Massachusetts, USA).

## Results

### General findings of the CS reconstruction

**Fig. 4:**
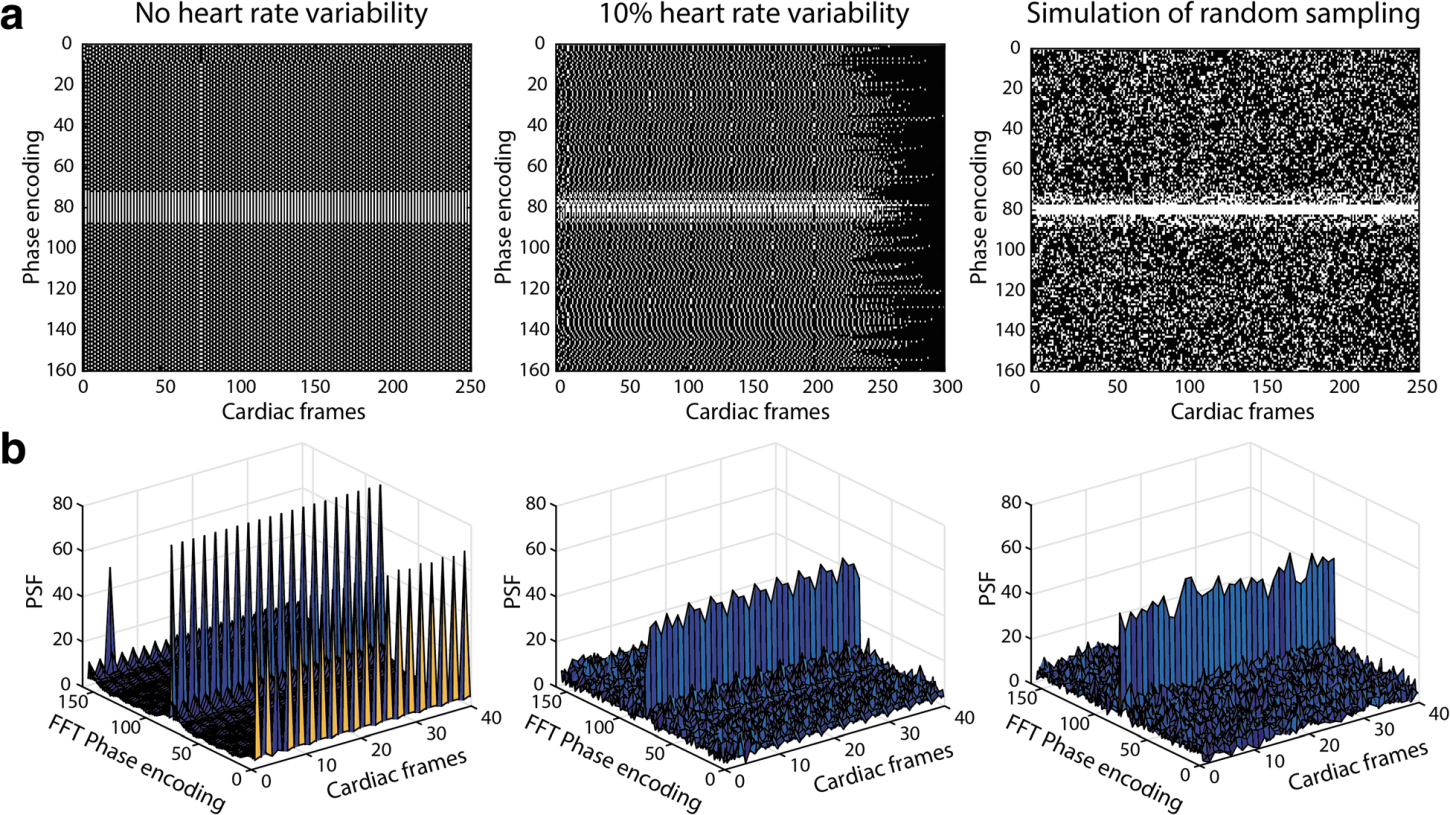
Different undersampling patterns and corresponding point spread function (PSF). **a** Binary masks of k_y_-t-space of phantom data at 0% heart rate variability (left) and 10% heart rate variability (middle). On the right is an ideal random sampling pattern with the same undersampling factor. **b** PSF after inverse fast fourier transform (FFT) in both spatial dimensions as function of time. The sampling pattern without heart rate variability (left) creates two side peaks, whereas the ideal random undersampling pattern (right) creates incoherent noise next to the main peak. A similar incoherent pattern is found with 10% heart rate variability (middle)

Figure 4a shows the undersampling pattern in k_y_-t-space of the retrospectively gated CMR sequence obtained from the phantom measurements with either 0% (left) or 10% (middle) heart rate variation, created by the flow phantom setup. Additionally, Fig. 4a, right shows a simulated, ideal incoherent sampling pattern. The respective point spread functions (PSFs = fast Fourier transforms (FFT) in x and y directions of the binary undersampling pattern) as a measure of incoherence are shown in Fig. 4b. For a perfectly regular heart rate (60 ± 0 bpm), the PSF had high side lobes at a frequency proportional to the undersampling factor (Fig. 4b, left). In contrast, a random heart rate led to incoherent sampling patterns and an improved PSF (Fig. 4b, middle), almost similar to the ideal simulated case (Fig. 4b, right).

**Fig. 5:**
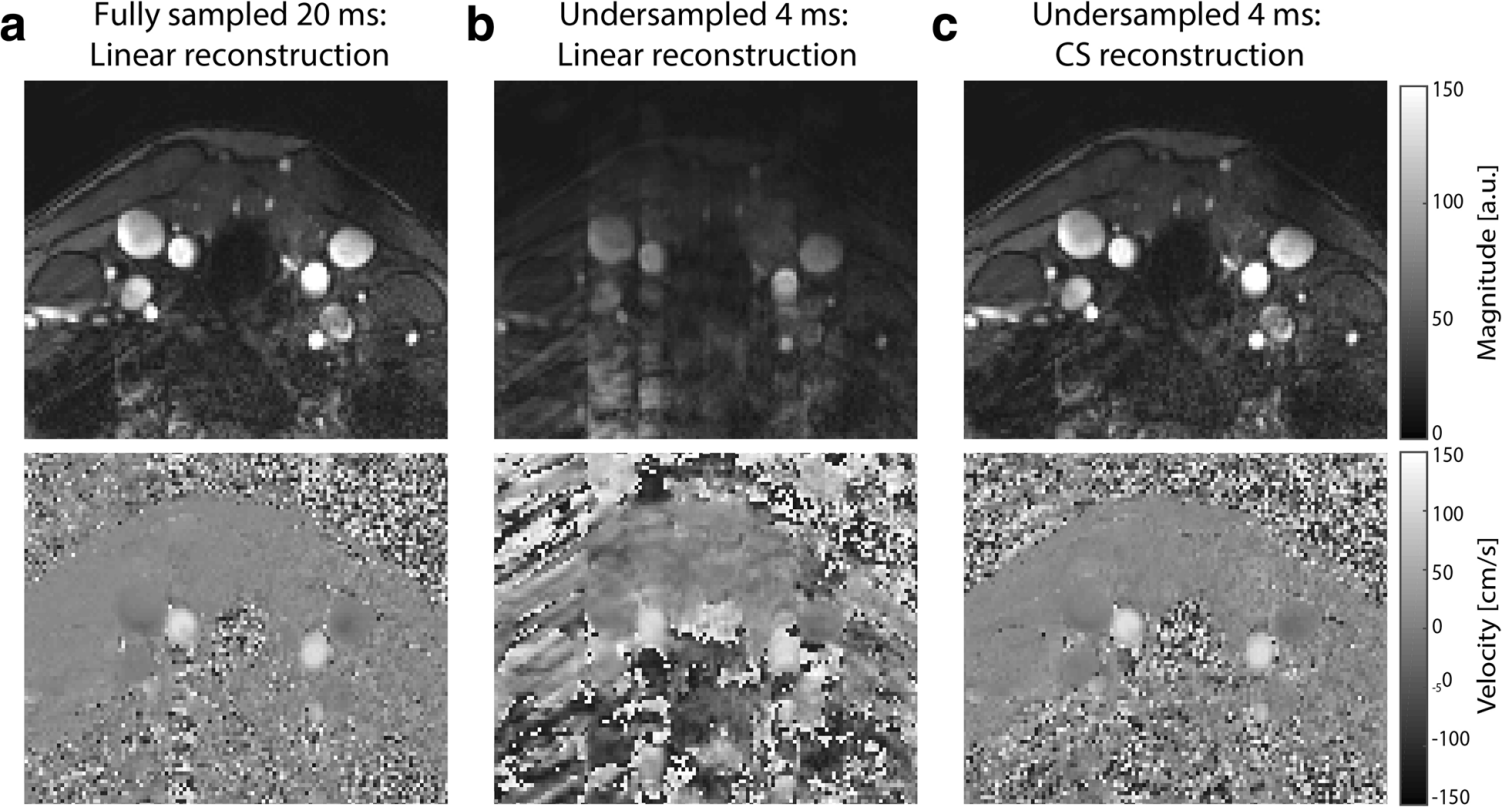
Linear and CS reconstructions of magnitude and velocity images. **a** Magnitude and velocity images from a fully sampled in vivo dataset of 20 ms temporal resolution. The same dataset reconstructed at a temporal resolution of 4 ms using (**b**) a linear reconstruction and (**c**) a compressed sensing (CS) reconstruction

**Fig. 6:**
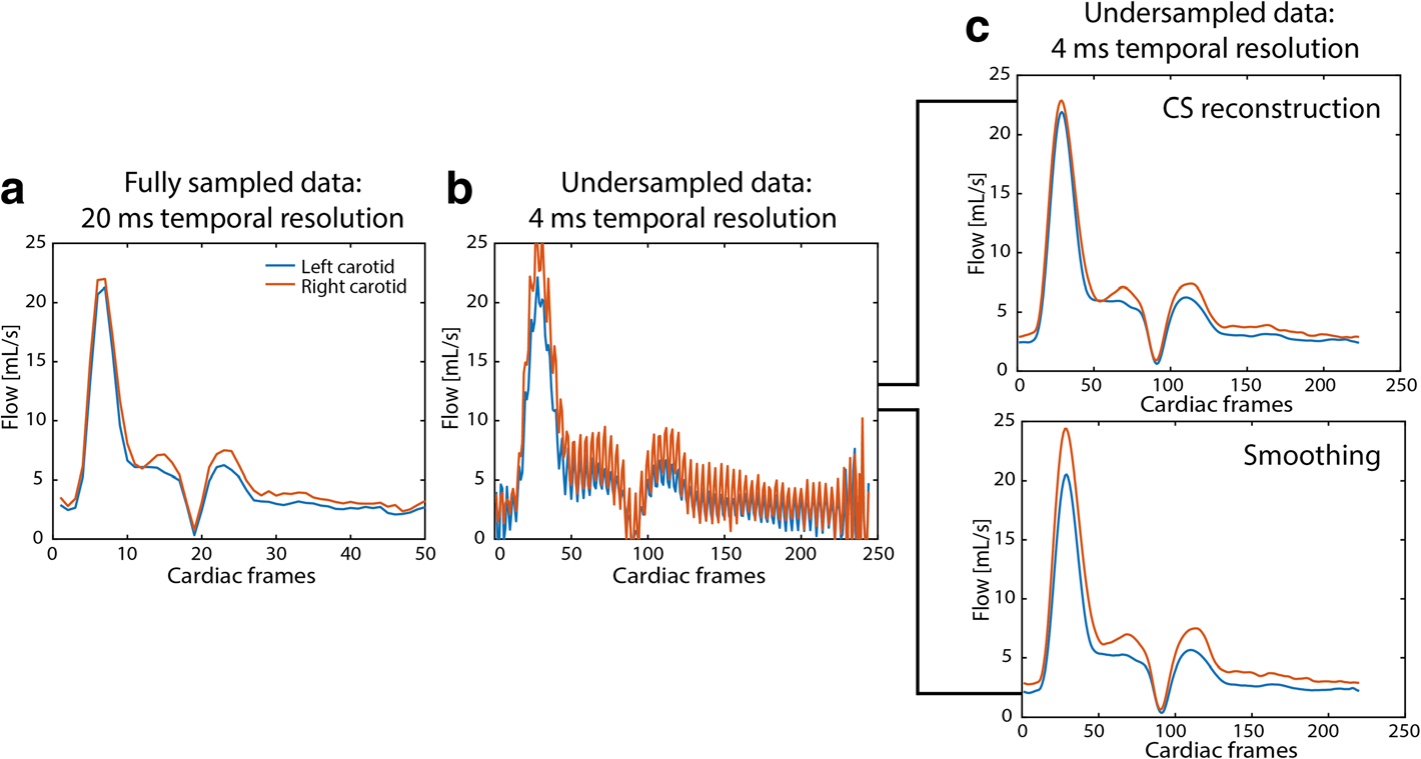
Comparison between smoothing and CS reconstruction. **a** Linear reconstruction of a fully sampled dataset (20 ms temporal resolution). **b** Linear reconstruction of undersampled data with 4 ms temporal resolution. **c** CS reconstruction recovering the undersampled data (top). Smoothing applied to the undersampled data without CS reconstruction (bottom) leads to incorrect reconstruction of the flow profile

The performance of the CS reconstruction was further demonstrated by the in vivo example in Fig. 5, where the high temporal resolution CS reconstruction of magnitude and phases images (Fig. 5c) exhibited the same image quality as for a fully sampled low temporal resolution scan (Fig. 5a). The corresponding flow curves of the fully sampled scan and the undersampled scan with a linear reconstruction are shown in Fig. 6a-b. Finally, Fig. 6c shows the difference between a CS reconstruction of the undersampled data as compared to performing a simple smoothing operation on the undersampled data. In case of smoothing, flow values were overestimated and did not compare to those determined from a fully sampled dataset. This demonstrated that temporal smoothing could not replace a CS reconstruction.

With a higher undersampling factor, i.e. a higher temporal resolution, undersampling artifacts got more prominent in the images when using a linear reconstruction, which was also visible in the flow curves (Additional file 2: Figure S2a, top row). On the other hand, after performing CS reconstruction, all flow curves were recovered (Additional file 2: Figure S2a, bottom row), showing the robustness of the method.

### Flow phantom experiments

**Fig. 7:**
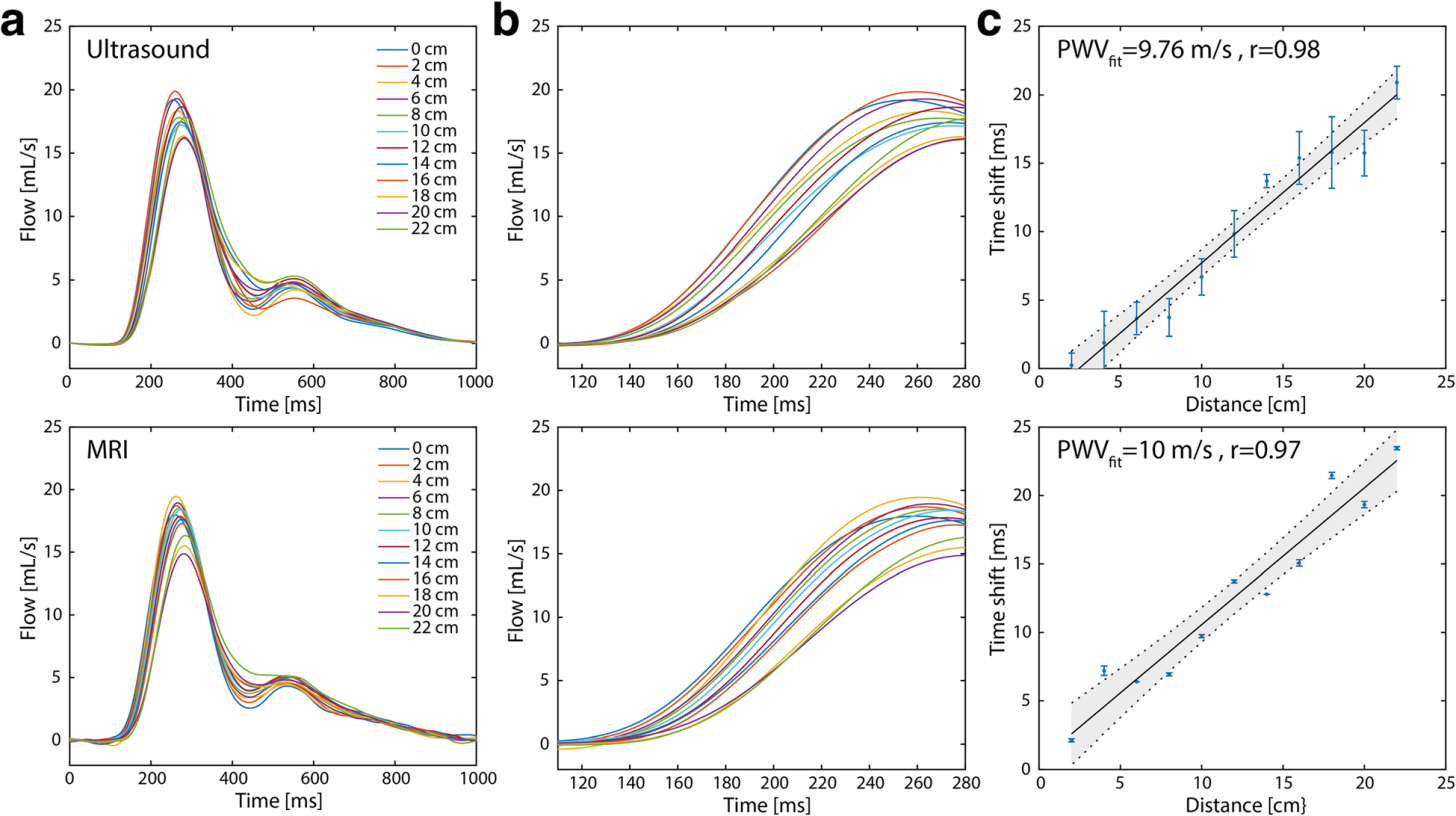
Ultrasound- and CMR-based PWV measurements in the flow phantom. **a** Flow in mL/s measured at 14 positions along the tube (every 2 cm) with the ultrasound probe (top) and with CMR (bottom). **b** Close-up of the region between 120 and 280 ms for better visibility of the time shifts between the curves. **c** Transit time versus the distance between the tubes. A linear fit yields the PWV

Flow curves measured at 12 positions along the phantom tube showed the expected time shifts along the tube both for ultrasound and MRI measurements (Fig. 7). Also, attenuation and broadening of the systolic flow peak towards the end of the tube was observed. Nevertheless, absolute flow values matched very well between ultrasound and CMR. By assessing the transit time as a function of the position along the tube, PWV could be derived from the slope of the linear relation (Fig. 7c). Error bars indicate the differences for different transit time calculation methods. Resulting PWV values were 9.8 ± 1.5 m/s and 10.0 ± 3.5 m/s for ultrasound and CMR measurements, respectively.

**Fig. 8:**
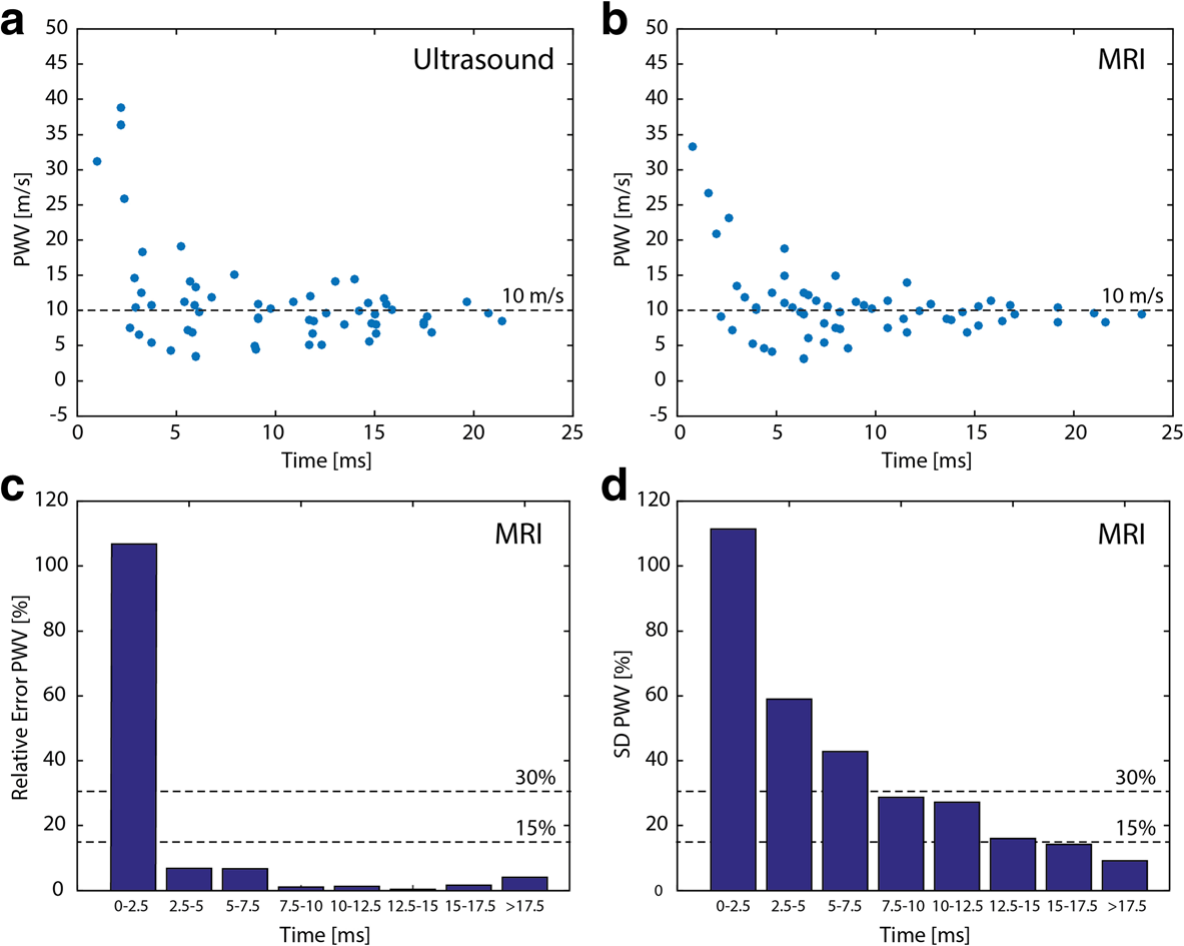
Accuracy and precision of PWVs from CMR. PWV values as function of the transit times in the phantom tube measured with (**a**) ultrasound and (**b**) CMR, calculated from all possible distance combinations. At shorter transit times (i.e. shorter distances) the PWV values are overestimated and variation is bigger. **c** Relative error of PWV values as function of transit time. For transit times shorter than 2.5 ms the mean PWV values deviate significantly from the expected value of 10 m/s. **d** Standard deviation (SD) of the mean PWV as function of transit time. For transit time above 7.5 ms, the standard deviation decreases below 30%

PWV values could additionally be obtained by considering all other combinations of measurement positions. In Fig. 8a-b**,** PWV values were plotted against the corresponding transit times. PWVs clearly became more inaccurate if the measurement sites were too close and therefore transit times were too short. In Fig. 8c-d, PWVs were grouped according to different transit time lengths, while showing the mean relative error in comparison to the expected PWV of 10 m/s and the standard deviation (SD) of PWV estimation, respectively. Only for the group with the smallest transit time, the mean error was much larger than 10%. SD values dropped below 30% for transit times larger than 7.5 ms. This may for example correspond to accurate PWV estimations up to 10 m/s for an arterial segment as small as 7.5 cm.

### In vivo experiments

**Fig. 9:**
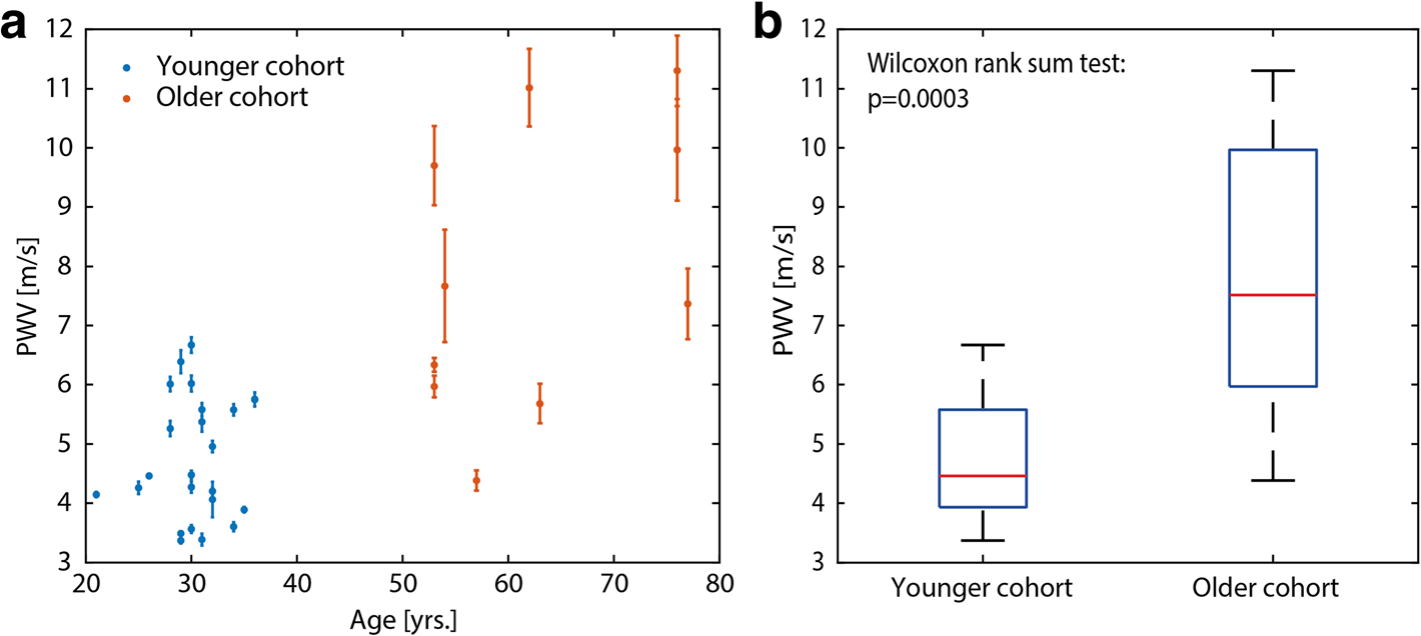
Association between PWV and age in vivo*.* PWV of healthy subjects plotted against age (**a**). The older cohort (red) has a significant higher PWV than the younger cohort (blue). **b** shows boxplot between the two groups. Error bars indicate one standard deviation resulting from calculating PWV with the 5 different methods

The mean carotid artery segment length as measured from the 3D black-blood scans was ∆x = 7.8 ± 1.4 cm. The mean transit time was ∆*t* = 15 ± 5 ms, with only 2 healthy subjects having transit times < 7.5 ms. Figure 9a shows PWV values plotted against age using data from all volunteers. Error bars indicate one standard deviation resulting from calculating PWV with the 5 different methods described previously (Additional file 1: Figure S1). Mean PWV for healthy young and elderly subjects were significantly different (4.7 ± 1.0 m/s and 7.9 ± 2.4 m/s, respectively; *p* < 0.001) as indicated in the boxplot of Fig. 9b.

## Discussion

PWV measurements within short carotid artery vessel segments require high spatiotemporal resolution flow data. This study demonstrates the feasibility and accuracy of PWV calculation based on 2D CINE PC CMR with retrospective gating, high temporal resolution binning and CS reconstruction.

Knowledge on regional PWV values in the carotid arteries may help in understanding the causes and consequences of stiffening and plaque development. The development of local vascular stiffening precedes atherosclerotic lesions before intima-media thickening as has been shown in mouse models [7]. However, the relation of PWV and atherosclerosis is still largely unknown in human, which is why further investigations on local PWVs are of high importance. Aortic wall stiffening and the ratio of aortic to carotid stiffness in particular, are implicated in cognitive decline [11]. Reduced PWV differences between aorta and carotid artery are associated with reduced blood flow volume towards the brain at older age [6]. Therefore, regional rather than global PWV measurements are needed to investigate PWV differences between different vessels. In the current form the regional PWV scan can be added to a standard clinical and research CMR protocol, which includes measurements of wall thickness, blood flow, and wall shear stress, to provide a comprehensive picture of vessel function.

Our proposed method was validated against ultrasound measurements showing that accurate PWV estimates can be achieved for transit times above 7.5 ms, with a deviation of mean PWV estimates < 5% and a SD of PWV estimates < 30%. This includes both deliberately introduced heart rate variations, as well as all variations caused during the measurement and analysis pipeline. Our measurements compete well with others [12, 21] reporting intra group SD variations of PWV values of 20% up to 40% for aortic segments which are generally longer and have lower PWV as compared to the carotids. Generally, the sampling frequency of the proposed method of 250 Hz can fully describe the flow curve, rather than a regular (prospectively gated) scan with a sampling frequency of 50 Hz. The frequency content of flow curves is mainly below 40 Hz [22]. Therefore, according to the Nyquist-Shannon rule, a sampling frequency of at least 80 Hz or more suffices to capture the full signal information. Besides achieving a sufficient temporal resolution, PWV estimation highly relies on the accuracy of the transit time calculation, which in turn depends on the accuracy the shape of the flow curves. These may be compromised by low SNR, imaging artifacts, or too high VENC values [23]. The relative PWV error decreases as the transit time is longer (either for higher PWVs or for longer vessel segments) as described for one-dimensional MRI technique Bolster et al. [21, 23]. Based on our current results, we therefore recommend to report a minimal transit time rather than a maximal PWV for this technique. Our data implies accurate PWV estimates of (up to) 10 m/s are possible in regional arterial segments down to 7.5 cm, which is shorter than reported for earlier carotid PWV strategies [7]. Moreover, we show that the systematic bias of our method is low, which is important when studying PWV differences between patient cohorts. Using error propagation one can estimate the PWV value accuracy. Assuming an error in spatial resolution of 0.8 mm and a temporal error of 50% of the temporal resolution, this would lead to a relative PWV error of 30% for 7.5 cm distance and a transit time of 7.5 ms, which agrees with our experimental findings.

The results of in vivo PWV measurements are in agreement with other reported values. Luo et al. measured PWV with ultrasound in the left carotid artery [24] in 8 male subjects (27 ± 4 years). PWV was found to be 4.5 ± 0.4 m/s on average. Wang et al. [25] measured an average PWV of 5.3 ± 0.7 m/s with ultrasound in 45 healthy subjects (25 male, mean ± SD age: 49 ± 3 years). Finally, a significant difference was shown for carotid PWV values between healthy young and elderly subjects, which agrees very well with age-related differences in carotid PWV reported by Kröner et al. [6] (PWV young 5.7 ± 1.0 m/s and PWV elderly 6.9 ± 1.5 m/s).

PWV inter-scan variation has been shown to be much larger than the intra-scan variation, not due to technical variability, but most likely caused by physiological variations [12]. The technique developed in this study was therefore validated against ultrasound measurements and by studying PWV differences between age groups.

Currently, Doppler ultrasound measurement of carotid-femoral PWV (global PWV) is considered the non-invasive gold standard for global arterial stiffness assessment [4]. Global PWV is calculated from the time shift between the pulse wave measured in the femoral artery and in the carotid artery divided by the distance between these points. However, global PWV has several limitations as it measures an average elasticity of a vascular system, which is known to be not uniform. Additionally, it is rather difficult to determine the distance between carotid and femoral artery accurately. In contrast, MRI provides flow values as well as detailed anatomical images, making the determination of the actual path length of the arterial segment and PWV calculations more accurate. Nonetheless, regional carotid ultrasound has certain advantages in that it is a fast and cost-efficient method. However, CMR additionally offers a range of imaging protocols to assess other aspects of atherosclerotic disease. Black-blood CMR can be used to accurately determine vessel wall thickness—a surrogate marker of atherosclerosis. With 4D flow CMR blood flow velocity and wall shear stress [26] can be derived. We therefore believe that a CMR-based regional PWV measurement will prove a valuable addition to a comprehensive vascular imaging examination.

In this study, the calculation of the transit time was based on an average value from several analysis methods, an approach which has also been suggested by others [2, 27] in order to avoid systematic errors in the transit time calculation. Based on our current results, we cannot conclude that one of the analysis methods is superior to the others, which further warrants this approach. Moreover, differences in PWV values between methods were much smaller than the differences between the two age groups.

Based on the dimension and material properties of the phantom tube, a PWV of 14.1 m/s was expected using the Moens-Korteweg equation [18], which is higher than what we measured experimentally. However, the tube was manually manufactured specifically for this experiment and inaccuracy in the reported elastic modulus (~ 0.5 GPa) cannot be excluded. Additionally, inflow effects at the inlet of the tube, as well as reflections at the outlet could have had an influence on the measured flow curves. Importantly though, both ultrasound and CMR agreed and measured a PWV of ~ 10 m/s.

A limiting factor to our method is that the 2D slices were acquired sequentially, which means that each slice was acquired during a different ensemble of heart beats. Although we cannot rule out that this may have introduced some variability, the effect is likely minimal because of the use of ‘absolute binning’ and the short acquisition time of only 3 min. An improvement to the current implementation would be a slice-interleaved acquisition or simultaneous multi-slice imaging [28]. Others have suggested the use of 4D flow CMR [29], facilitating flow curves along the entire vessel path. However, at this moment this method would result in clinically unfeasible examination times when combined with the spatiotemporal resolution needed for carotid artery imaging.

## Conclusions

We demonstrate that retrospective gating, undersampling, and a CS reconstruction allow for high temporal resolution 2D CINE PC CMR, enabling accurate and regional carotid PWV quantification. This technique may prove a valuable addition to a comprehensive vascular imaging examination, which addresses various aspects of atherosclerotic disease.

## Additional files

Additional file 1: **Figure S1.** Overview of methods used to calculate transit time between two flow curves. Calculation of the transit time from the two flow curves using (a) the CC method and (b) the FF method. (c) Calculation of transit time using the FF method in combination with a normalization of the flow curves. Calculation of the transit time fitting a sigmoid function through the flow curves for (d) CC and (e) FF method. (JPG 655 kb)

Additional file 2: **Figure S2.** Flow curves with different temporal resolutions. The same data of one exemplary in vivo dataset is binned at a temporal resolution of 10, 8 and 4 ms (from left to right), resulting in 100, 120 and 250 frames across the cardiac cycle. Binning the data at a higher temporal resolution than in 20 ms leads to undersampling. (a) Flow curves derived from a linear image reconstruction with increasing undersampling artifacts at higher undersampling factors. (b) Flow curves after CS reconstruction. (JPG 1390 kb)

## Abbreviations

2D: Two dimensional
CC: Cross-correlation method
CCA: Common carotid artery
CMR: Cardiovascular magnetic resonance
CS: Compressed sensing
ECG: Electrocardiogram
FA: Flip angle
FF: Foot-to-foot method
FFE: Fast field echo
FFT: Fast Fourier transform
FH: Foot-head direction
FOV: Field of view
PC: Phase contrast
PROUD: Scanner patch to PROspective Undersampling in multiple Dimensions
PSF: Point spread function
PWV: Pulse wave velocity
ROI: region of interest
SD: Standard deviation
TE: Echo time
TR: Repetition time
TV: Total variation
VENC: Velocity encoding gradient
WSS: Wall shear stress

## References

[1] Coolen BF, Calcagno C, van Ooij P, Fayad ZA, Strijkers GJ, Nederveen AJ (2017). Vessel wall characterization using quantitative MRI: what’s in a number?. Magn Reson Mater Physics, Biol Med Springer Berlin Heidelberg.

[2] Wentland AL, Grist TM, Wieben O (2014). Review of MRI-based measurements of pulse wave velocity: a biomarker of arterial stiffness. Cardiovasc Diagn Ther.

[3] van Popele NM, Grobbee DE, Bots ML, Asmar R, Topouchian J, Reneman RS (2001). Association between arterial stiffness and atherosclerosis. Stroke.

[4] Pereira T, Correia C, Cardoso J (2015). Novel methods for pulse wave velocity measurement. J Med Biol Eng.

[5] Rogers WJ, Hu Y, Coast D, Vido DA, Kramer CM, Pyeritz RE (2001). Age-associated changes in regional aortic pulse wave velocity. J Am Coll Cardiol.

[6] Kröner ESJ, Lamb HJ, Siebelink HMJ, Cannegieter SC, van den Boogaard PJ, van der Wall EE (2014). Pulse wave velocity and flow in the carotid artery versus the aortic arch: effects of aging. J Magn Reson Imaging.

[7] Gotschy A, Bauer E, Schrodt C, Lykowsky G, Ye YX, Rommel E (2013). Local arterial stiffening assessed by MRI precedes atherosclerotic plaque formation. Circ Cardiovasc Imaging.

[8] Bos D, Portegies ML, van der Lugt A, Bos MJ, Koudstaal PJ, Hofman A (2014). Intracranial carotid artery atherosclerosis and the risk of stroke in whites The Rotterdam Study. JAMA Neurol.

[9] Truijman MTB, Kooi ME, van Dijk AC, de Rotte AAJ, van der Kolk AG, Liem MI (2014). Plaque at RISK (PARISK): prospective multicenter study to improve diagnosis of high-risk carotid plaques. Int J Stroke.

[10] Gotschy A, Bauer WR, Winter P, Nordbeck P, Rommel E, Jakob PM (2017). Local versus global aortic pulse wave velocity in early atherosclerosis: an animal study in ApoE−/−−mice using ultrahigh field MRI. PLoS One.

[11] de Roos A, van der Grond J, Mitchell G, Westenberg J (2017). Magnetic resonance imaging of cardiovascular function and the brain—is dementia a cardiovascular-driven disease?. Circulation.

[12] Markl M, Wallis W, Strecker C, Gladstone BP, Vach W, Harloff A (2012). Analysis of pulse wave velocity in the thoracic aorta by flow-sensitive four-dimensional MRI: reproducibility and correlation with characteristics in patients with aortic atherosclerosis. J Magn Reson Imaging.

[13] Coolen BF, Abdurrachim D, Motaal AG, Nicolay K, Prompers JJ, Strijkers GJ (2013). High frame rate retrospectively triggered Cine MRI for assessment of murine diastolic function. Magn Reson Med.

[14] Lustig M, Donoho D, Pauly JM (2007). Sparse MRI: the application of compressed sensing for rapid MR imaging. Magn Reson Med.

[15] Tran-Gia J, Koestler H, Seiberlich N, Imaging F. In: Syed MA, Raman SV, Simonetti OP, editors. Basic Princ Cardiovasc MRI Phys imaging tech. Switzerland: Springer International Publishing; 2015. p. 69–70.

[16] Uecker M, Ong F, Tamir JI, Bahri D, Virtue P, Cheng JY, et al. Berkeley advanced reconstruction toolbox. Proc Intl Soc Mag Reson Med. 2015:2486.

[17] Rosset A, Spadola L, Ratib O (2004). OsiriX: an open-source software for navigating in multidimensional DICOM images. J Digit Imaging.

[18] Vlachopoulos C, O’Rourke M, Nichols WW. McDonald’s blood flow in arteries. Boca Raton: CRC Press; 2011.

[19] Fielden SW, Fornwalt BK, Jerosch-herold M, Eisner RL, Stillman AE, Oshinski JN (2008). A new method for the determination of aortic pulse wave velocity using cross-correlation on 2D PCMR velocity data. J Magn Reson Imaging.

[20] Wang J, Yarnykh VL, Yuan C (2010). Enhanced image quality in black-blood MRI using the improved motion-sensitized driven-equilibrium (iMSDE) sequence. J Magn Reson Imaging.

[21] Noda C, Ambale Venkatesh B, Ohyama Y, Liu CY, Chamera E, Redheuil A (2016). Reproducibility of functional aortic analysis using magnetic resonance imaging: the MESA. Eur Heart J Cardiovasc Imaging.

[22] Muñoz-Tsorrero JFS, Tardio-Fernandez M, Valverde-Valverde JM, Duque-Carrillo F, Vega-Fernandez JM, Joya-Vazquez P (2014). Pulse wave velocity in four extremities for assessing cardiovascular risk using a new device. J Clin Hypertens.

[23] Bolster BD, Atalar E, Hardy CJ, McVeigh ER (1998). Accuracy of arterial pulse-wave velocity measurement using MR. J Magn Reson Imaging.

[24] Luo J, Li RX, Konofagou EE (2012). Pulse wave imaging of the human carotid artery: an in vivo feasibility study. IEEE Trans Ultrason Ferroelectr Freq Control.

[25] Wang Z, Yang Y, Yuan L, Liu J, Duan Y, Cao T. Noninvasive method for measuring local pulse wave velocity by dual pulse wave Doppler: in vitro and in vivo studies. PLoS One. 2015;10:e0120482. 10.1371/journal.pone.0120482 PMC4364771 25786124

[26] Potters WV, Marquering HA, VanBavel E, Nederveen AJ (2014). Measuring wall shear stress using velocity-encoded MRI. Curr Cardiovasc Imaging Rep.

[27] Dyverfeldt P, Ebbers T, Länne T (2014). Pulse wave velocity with 4D flow MRI: systematic differences and age-related regional vascular stiffness. Magn Reson Imaging.

[28] Jin N, Pang J, Giri S, Speier P, Wang D. Simultaneous multi-slice phase contrast imaging for pulse wave velocity measurement in the vessel. Proc Intl Soc Mag Reson Med. 2017:1265.

[29] Markl M, Frydrychowicz A, Kozerke S, Hope M, Wieben O (2012). 4D flow MRI. J Magn Reson Imaging.

