# Supplementary figures and images for: Regional assessment of carotid artery pulse wave velocity using compressed sensing accelerated high temporal resolution 2D CINE phase contrast cardiovascular magnetic resonance
Source: J Cardiovasc Magn Reson. 2018 Dec 20;20:86. doi: 10.1186/s12968-018-0499-y (PMC6300923; doi:10.1186/s12968-018-0499-y)

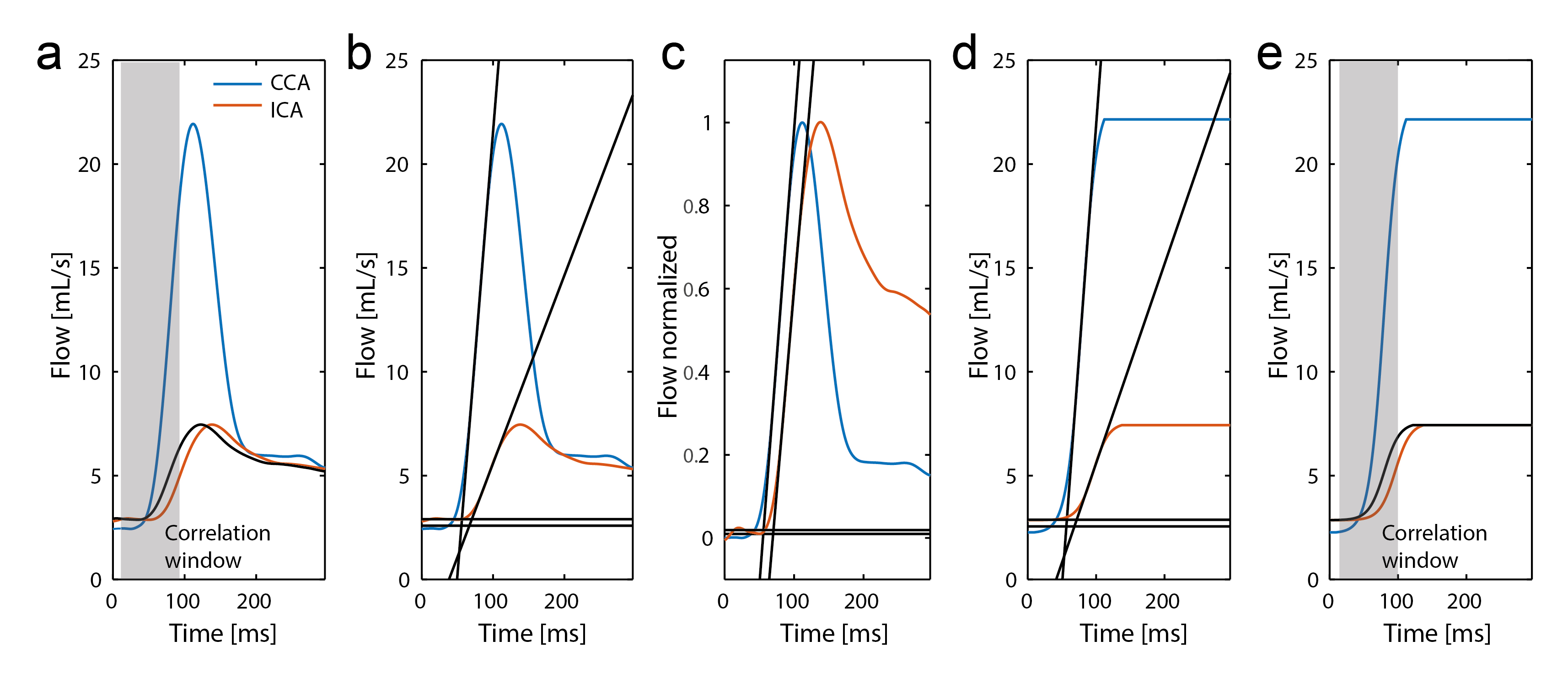

Supplement: Supplementary file 1 — Figure S1. Overview of methods used to calculate transit time between two flow curves. Calculation of the transit time from the two flow curves using (a) the CC method and (b) the FF method. (c) Calculation of transit time using the FF method in combination with a normalization of the flow curves. Calculation of the transit time fitting a sigmoid function through the flow curves for (d) CC and (e) FF method. (JPG 655 kb) [file 12968_2018_499_MOESM1_ESM.jpg]

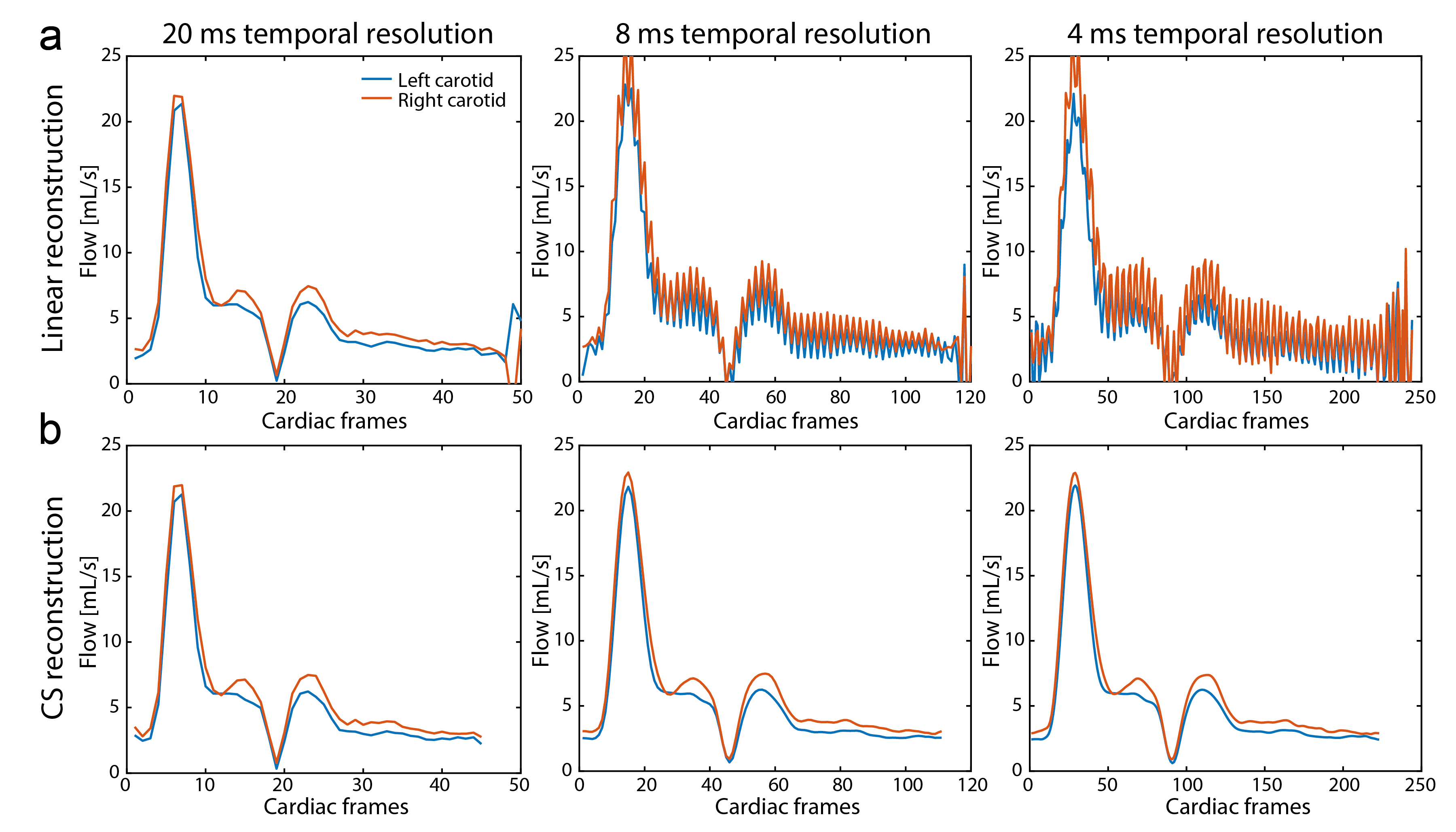

Supplement: Supplementary file 2 — Figure S2. Flow curves with different temporal resolutions. The same data of one exemplary in vivo dataset is binned at a temporal resolution of 10, 8 and 4 ms (from left to right), resulting in 100, 120 and 250 frames across the cardiac cycle. Binning the data at a higher temporal resolution than in 20 ms leads to undersampling. (a) Flow curves derived from a linear image reconstruction with increasing undersampling artifacts at higher undersampling factors. (b) Flow curves after CS reconstruction. (JPG 1390 kb) [file 12968_2018_499_MOESM2_ESM.jpg]
